# 149. Extraction-free RT-PCR to Detect SARS-CoV-2 Variants of Concern

**DOI:** 10.1093/ofid/ofab466.149

**Published:** 2021-12-04

**Authors:** Brian L Harry, Yue Qiu, Ling Lu, Mara Couto-Rodriguez, Dorottya Nagy-Szakal, Niamh B O’Hara, Shi-Long Lu

**Affiliations:** 1 University of Colorado, Aurora, CO; 2 Biotia, New York, New York

## Abstract

**Background:**

SARS-CoV-2 variants of concern (VOC) have challenged real-time reverse transcriptase polymerase chain reaction (RT-PCR) methods for the diagnosis of COVID-19.

**Methods:**

The CDC 2019-Novel Coronavirus real-time RT-PCR panel was modified to create a single-plex extraction-free proxy RT-PCR assay, VOCFast™. This assay uses the nucleocapsid N1 as well as novel primer/probe pairs to target VOC mutations in the Orf1a and spike (S) genes. For analytical validation of VOCFast, synthetic controls for the Wuhan, alpha/B.1.1.7, beta/B.1.351, and gamma/P.1 strains were tested at various concentrations. Clinical validation was performed using patient anterior nares swab and saliva specimens collected in the Denver, CO area between Nov 2020 and Feb 2021 or in March 2021. Orthogonal next-generation sequencing (NGS) was also performed.

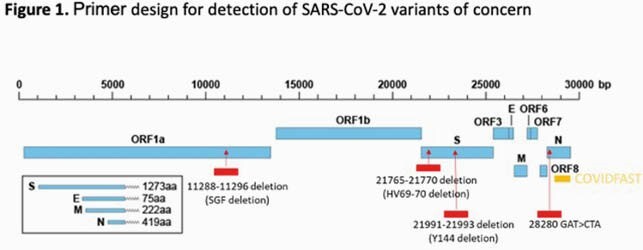

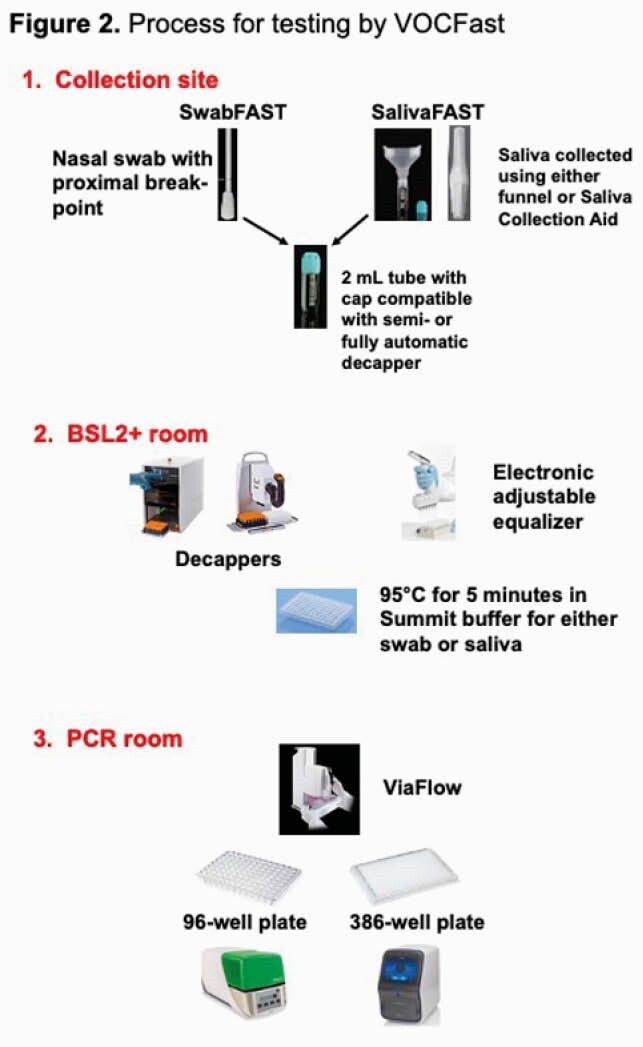

**Results:**

Similar N1 quantification cycle (Cq) values corresponding to viral load were observed for all strains, suggesting that VOC mutations do not affect performance of the N1 primer/probe. Orf1a-mut and S1-mut primer/probes generated a stable high Cq value for the Wuhan strain. Conversely, Orf1a-mut Cq values were inversely correlated with viral load for all VOC. The S1-mut Cq was inversely correlated with viral load of the alpha strain, but did not reliably amplify beta/gamma VOC. The limit of detection was 8 copies/uL.

The first set of COVID-19 patient specimens revealed no amplification using Orf1a-mut whereas 53% of specimens collected in Mar 2021 demonstrated amplification by Orf-1a. Orthogonal testing by the SARS-CoV-2 NGS Assay and COVID-DX software demonstrated that 12/12 alpha strains, 2/2 beta/gamma strains, and 33/33 Wuhan strains were correctly identified by VOCFast.

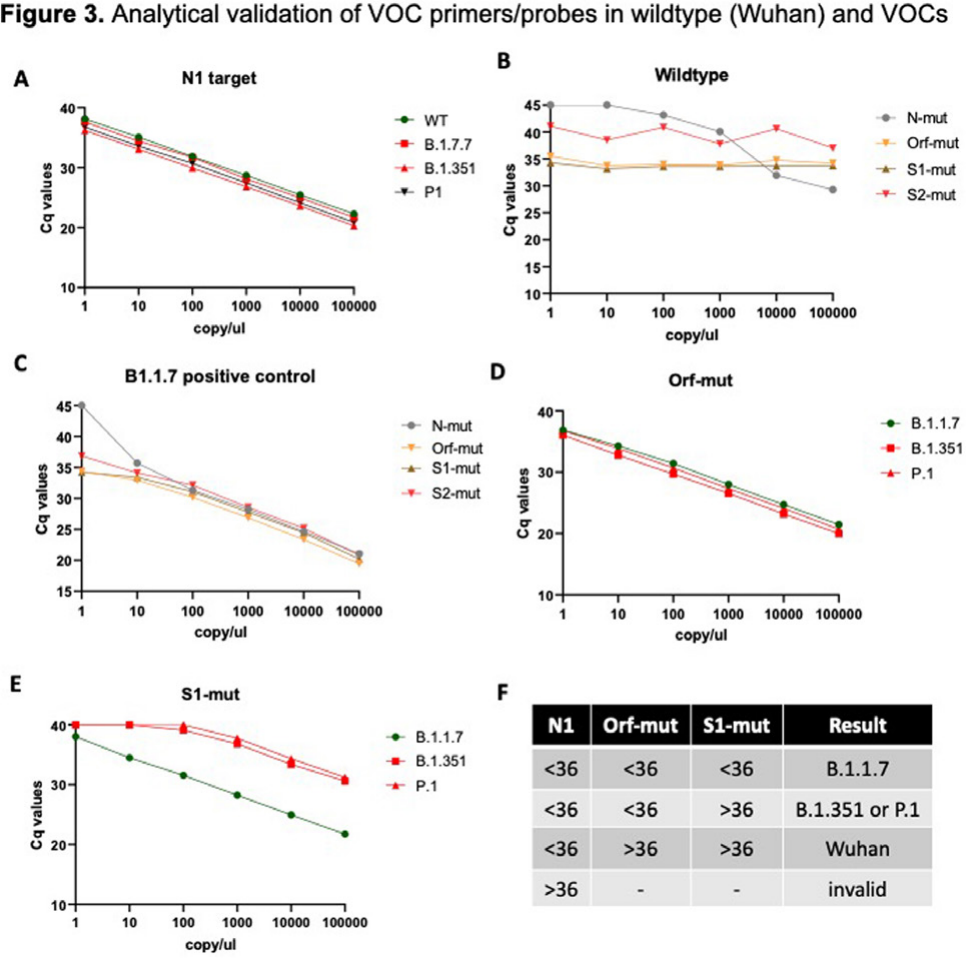

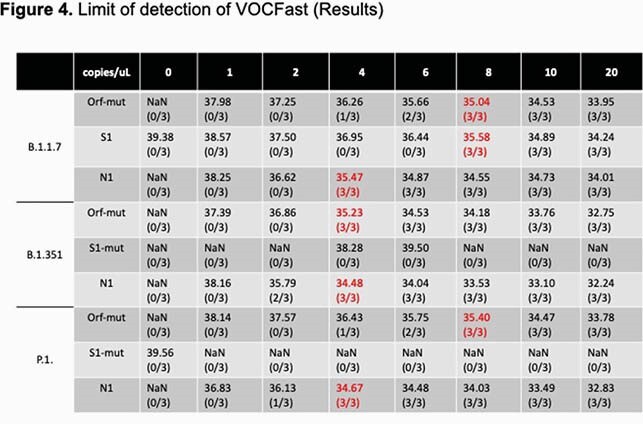

Detection of VOC in clinical specimens and validation by NGS

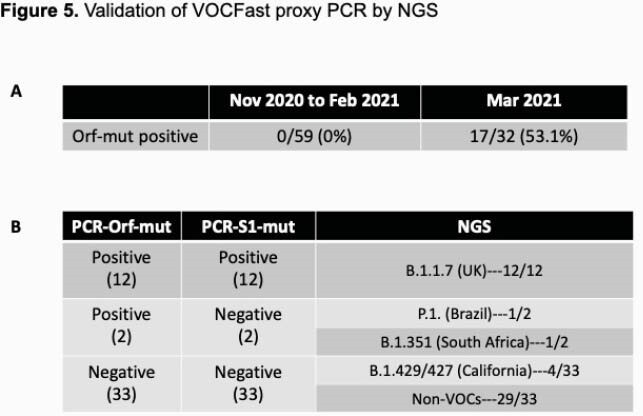

**Conclusion:**

The combination of the N1, Orf1a-mut, and S1-mut primers/probes in VOCFast can distinguish the Wuhan, alpha, and beta/gamma strains and it consistent with NGS results. Testing of clinical samples revealed that VOC emerged in Denver, CO in March 2021. Future work to discriminate beta, gamma, and emerging VOC is ongoing. In summary, VOCFast is an extraction-free RT-PCR assay for nasal swab and saliva specimens that can identify VOC with a turnaround time suitable for clinical testing.

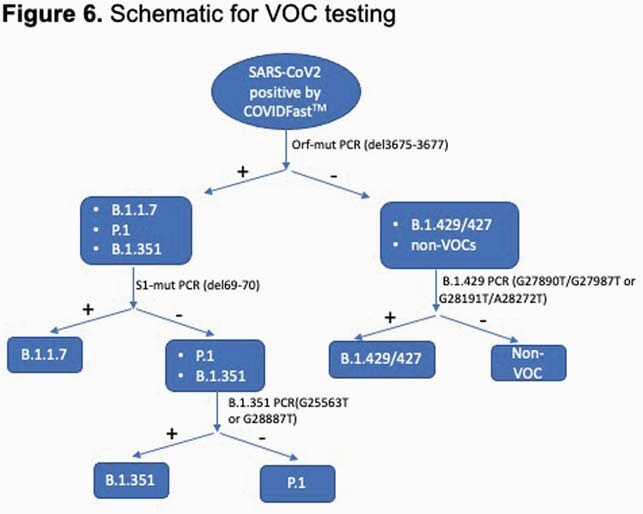

**Disclosures:**

**Brian L. Harry, MD PhD**, **Summit Biolabs Inc.** (Grant/Research Support, Shareholder) **Mara Couto-Rodriguez, MS**, **Biotia** (Employee) **Dorottya Nagy-Szakal, MD PhD**, **Biotia Inc** (Employee, Shareholder) **Niamh B. O’Hara, PhD**, **Biotia** (Board Member, Employee, Shareholder) **Shi-Long Lu, MD PhD**, **Summit Biolabs Inc.** (Grant/Research Support, Shareholder)

